# Impact of emergency nursing interventions on pre-hospital and in-hospital outcomes in acute ischemic stroke

**DOI:** 10.3389/fneur.2025.1673515

**Published:** 2025-11-24

**Authors:** Tingting Zhan, Chunyan Xu

**Affiliations:** 1 Department of Emergency, Wuhan Fourth Hospital, Wuhan, China; 2 Department of Neurology, Wuhan Fourth Hospital, Wuhan, China

**Keywords:** acute ischemic stroke, emergency nursing, stroke code activation, door-to-needle time, pre-hospital care, neurological outcome, thrombolysis, retrospective cohort

## Abstract

**Objective:**

Acute ischemic stroke (AIS) is a leading cause of mortality and long-term disability worldwide, with clinical outcomes highly dependent on the timeliness and coordination of emergency care. Emergency nurses are at the frontline of acute stroke management, contributing significantly to early recognition, rapid triage, thrombolysis preparation, and prevention of in-hospital complications. However, the real-world impact of structured emergency nursing interventions on both pre-hospital and in-hospital stroke outcomes remains underexplored. This study aimed to evaluate the feasibility and clinical effectiveness of emergency nursing interventions in reducing treatment delays and improving short-term neurological recovery in patients with AIS.

**Methods:**

A retrospective cohort study was conducted at Wuhan Fourth Hospital, including 217 adult AIS patients admitted between January 2020 and April 2024. Patients were divided into two groups based on the presence or absence of structured emergency nursing protocols, including pre-hospital triage coordination, stroke code activation, focused neurological monitoring, and post-thrombolysis care. Primary endpoints included door-to-needle time (DNT), thrombolysis rate, and early neurological deterioration. Secondary outcomes were NIHSS score changes at 72 h, hospital length of stay, and 7-day in-hospital mortality.

**Results:**

Patients receiving emergency nursing interventions (*n =* 107) had significantly shorter median DNT (42 vs. 56 min, *p* < 0.001), higher thrombolysis rates (71.0% vs. 51.4%, *p* = 0.004), and reduced early neurological deterioration (10.3% vs. 21.5%, *p* = 0.018). NIHSS improvement ≥4 points was more frequent in the intervention group (64.5% vs. 43.1%, *p* = 0.003). No significant difference in 7-day mortality was observed.

**Conclusion:**

The implementation of structured emergency nursing interventions in AIS care significantly improves treatment timeliness and short-term functional outcomes. These findings support the inclusion of specialized nursing protocols in emergency stroke pathways to enhance quality and efficiency of care.

## Introduction

Acute ischemic stroke (AIS) remains a leading cause of death and long-term disability globally, contributing substantially to the burden of neurological disease and posing critical challenges to healthcare systems (1–3). The cornerstone of effective AIS management lies in the timely restoration of cerebral perfusion, particularly through intravenous thrombolysis and mechanical thrombectomy. However, the success of these time-sensitive interventions is heavily dependent on the efficiency of early recognition, rapid triage, and coordinated in-hospital workflows. The widely accepted principle of “time is brain” emphasizes that delays in initiating treatment—whether at the pre-hospital or emergency department level—can drastically reduce the likelihood of favorable neurological recovery, with each passing minute resulting in irreversible neuronal loss (4–6).

Emergency nursing staff are often the first point of clinical contact for patients presenting with acute stroke symptoms (7, 8). Their responsibilities extend from early recognition and rapid pre-hospital notification to the activation of stroke response pathways and coordination of diagnostic and therapeutic processes upon hospital arrival. In addition to clinical surveillance and vital sign monitoring, nurses are also instrumental in facilitating rapid imaging (such as CT scans), ensuring timely blood sampling and laboratory results, administering thrombolytic agents, and continuously evaluating for early signs of neurological deterioration or treatment complications. Despite their pivotal role, the specific impact of emergency nursing interventions—particularly standardized nursing protocols—on pre-hospital and in-hospital outcomes in AIS has not been adequately quantified in real-world clinical practice (9–11). In recent years, stroke care systems have increasingly emphasized structured multidisciplinary collaboration, yet the integration of nursing-led initiatives remains uneven across institutions. There is a growing need to evaluate how emergency nursing practices, including stroke-specific triage tools, pre-hospital education, and thrombolysis preparation protocols, affect key performance metrics such as onset-to-door time, door-to-needle time (DNT), and early patient outcomes (12, 13). This is particularly relevant in resource-constrained settings, where optimizing human workflow efficiency can significantly influence treatment rates and reduce the burden of long-term disability.

Therefore, this retrospective study aimed to examine the feasibility and effectiveness of emergency nursing interventions in AIS patients managed at Wuhan Fourth Hospital between January 2020 and April 2024. We specifically evaluated the influence of structured nursing actions on treatment timelines, thrombolysis initiation rates, and short-term clinical outcomes, with the goal of providing evidence to support scalable, nurse-led models of stroke emergency care.

## Materials and methods

### Study design and participants

This retrospective cohort study was conducted in the Emergency Department of Wuhan Fourth Hospital, spanning the period from January 2020 to April 2024. The objective was to evaluate the effect of structured emergency nursing interventions on the clinical outcomes of patients diagnosed with acute ischemic stroke (AIS). A total of 217 adult patients (aged ≥18 years) with imaging-confirmed AIS were enrolled. Eligibility criteria included symptom onset within 24 h prior to admission, confirmed diagnosis by CT or MRI, and availability of complete pre-hospital and in-hospital clinical data. Patients were excluded if they had hemorrhagic stroke, stroke mimics (such as hypoglycemia or seizures), or pre-existing severe disability (modified Rankin Scale ≥4). Based on the type of emergency care received, patients were assigned to either the intervention group (*n =* 109), which received structured emergency nursing management, or the standard care group (*n =* 108), which received standard emergency care. Routine COVID-19 screening was performed for all patients upon hospital arrival in accordance with institutional infection-control policies. These procedures were implemented in parallel with stroke triage and treatment, and did not affect the stroke care pathway or the nursing interventions analyzed in this study.

Imaging was reviewed by a senior neurologist and a radiologist. The radiologist performed the initial analysis of CT or MRI scans to identify contraindications for thrombolysis. After the radiologist’s review, the neurologist conducted a further examination of the imaging along with clinical data to make the final decision regarding thrombolysis eligibility and treatment initiation.

### Stroke care in the region

In our region, stroke care is organized through an integrated emergency medical service (EMS) and hospital-based stroke unit network. Suspected stroke patients are typically identified and transported by EMS directly to the emergency department (ED) of designated stroke centers. Upon arrival, ED nurses conduct the initial triage, including stroke symptom screening and vital sign assessment, before immediate referral to the stroke team for neuroimaging and treatment decisions. Patients eligible for thrombolysis receive treatment in the ED and are subsequently admitted to the stroke unit or intensive care unit for further monitoring. This framework provides the standard of care against which the structured nurse-led stroke pathway was implemented in this study.

### Emergency nursing intervention protocol

The standard emergency team consisted of emergency physicians, neurologists, nurses, and radiologists. Emergency physicians conducted the initial assessment and stabilization of patients, while neurologists provided specialized consultations for stroke management. Nurses, including those involved in the intervention group, performed initial assessments, monitored symptoms, and delivered supportive care under the supervision of the neurologist. The team worked collaboratively in the emergency department and designated stroke units.

The intervention group received comprehensive stroke-focused nursing care from trained emergency nurses following a standardized protocol. Upon pre-hospital identification of stroke symptoms (typically via the FAST screening tool), EMS personnel notified the emergency department in advance to initiate stroke team preparation. Upon arrival, triage nurses expedited patient registration and prioritized vital sign stabilization, intravenous line placement, and blood collection. A rapid neurological assessment was conducted using the National Institutes of Health Stroke Scale (NIHSS). The stroke nursing team coordinated prompt neuroimaging and collaborated with the stroke neurologist to assess thrombolysis eligibility. For thrombolysis candidates, nurses administered alteplase, monitored infusion rates and vital parameters, and recorded time intervals, including door-to-needle and door-to-CT times. Continuous monitoring was conducted throughout the emergency stay, and nursing documentation included real-time logging of symptoms, assessments, and adverse events. Thrombolysis was administered to eligible patients in the emergency department (ED) based on clinical assessments and imaging results. All patients who received thrombolysis were admitted to the hospital for further monitoring. Depending on their condition, patients were either admitted to the stroke unit or the intensive care unit (ICU) for close monitoring after thrombolysis. Both the intervention and standard care groups followed the same protocol for thrombolysis administration, ensuring consistency across patient groups. In contrast, the standard care group received routine emergency care without specialized stroke nursing protocols, and their care adhered to standard triage and treatment practices. Standard care groupThe nursing interventions were supervised by neurologists to ensure adherence to established stroke care protocols. Neurologists were not always physically present but provided remote supervision. They were actively involved through telecommunication platforms, reviewing clinical assessments, patient histories, and initial evaluation results. This allowed real-time feedback to nursing staff to ensure proper protocol adherence. Critical decisions regarding thrombolysis and other emergency interventions were made after review by a neurologist, who approved the treatment plan.

In the standard care group, patients received routine emergency management according to national stroke guidelines, including EMS transport, initial triage in the emergency department, physician-led neurological evaluation, and general nursing support without structured protocols. Prehospital stroke recognition relied mainly on routine EMS assessments and family-initiated calls. In contrast, the nurse-led intervention group incorporated additional structured components: (1) community education on stroke recognition delivered by nurses; (2) targeted EMS staff training by stroke nurses to improve symptom identification and enable pre-hospital notification; (3) nurse-led triage in the emergency department with prioritized coordination for neuroimaging; and (4) continuous monitoring and documentation of critical treatment time intervals (Supplementary Table 1). These measures were designed to enhance early recognition, streamline workflows, and reduce delays across the prehospital and in-hospital care continuum.

### Data collection and outcome measures

All data were collected from the hospital’s electronic medical record system and pre-hospital EMS database. Primary outcomes included door-to-CT time, door-to-needle time (for thrombolyzed patients), the proportion of patients who received intravenous thrombolysis, and early neurological improvement defined as a ≥ 4-point reduction in NIHSS score within 72 h. Secondary outcomes included length of stay in the emergency department and hospital, rate of in-hospital complications (including aspiration pneumonia, symptomatic intracranial hemorrhage, and urinary tract infection), and modified Rankin Scale at discharge. Additional data on patient demographics, stroke risk factors, baseline NIHSS score, and time from symptom onset to hospital arrival were also collected.

### Statistical analysis

Data were analyzed using SPSS version 26.0 (IBM Corp., Armonk, NY, USA). Continuous variables were expressed as mean ± standard deviation or median with interquartile range, and were compared using independent-samples *t*-tests or Mann–Whitney U tests, depending on data distribution. Categorical variables were presented as frequencies and percentages, and compared using chi-square or Fisher’s exact tests. To assess the independent effect of emergency nursing intervention on favorable outcomes, multivariate logistic regression analysis was performed, adjusting for age, sex, baseline NIHSS score, comorbidities, and time from symptom onset to hospital arrival. A two-tailed *p* < 0.05 was considered statistically significant. Multivariate logistic regression analysis was used to identify independent predictors of early thrombolysis and favorable clinical outcomes. Model calibration was assessed using the Hosmer–Lemeshow goodness-of-fit test, with *p* > 0.05 indicating adequate model fit.

## Results

### Baseline characteristics of participants

**Figure 1:**
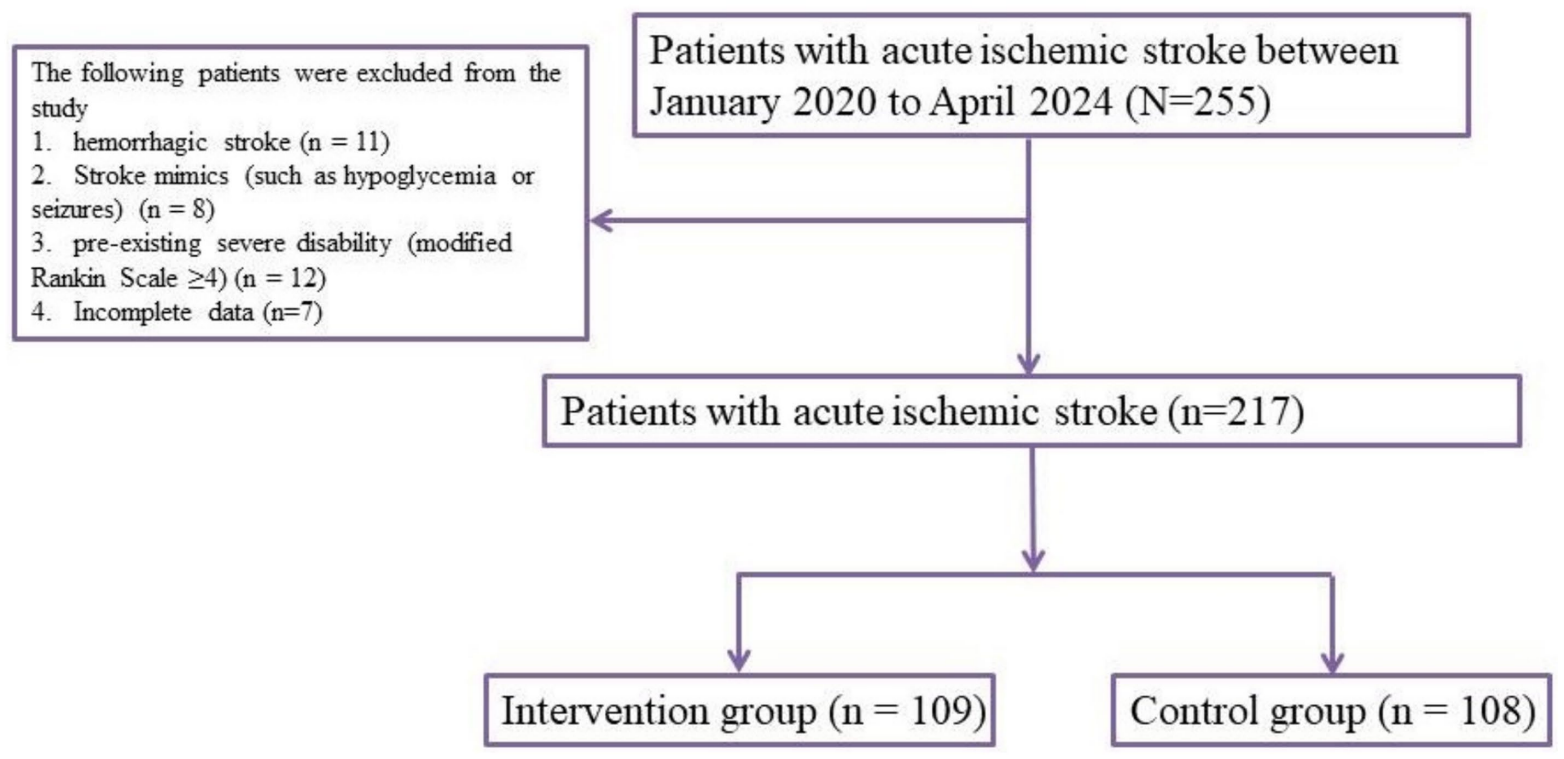
Flowchart of patient selection and group allocation.

A total of 217 patients with acute ischemic stroke were included in the analysis, with 109 patients receiving structured emergency nursing intervention (intervention group) and 108 patients receiving standard emergency care (Figure 1). Baseline demographic and clinical characteristics between the two groups were largely comparable. The mean age of the intervention group was 67.2 ± 9.4 years, and 65.8 ± 10.1 years in the standard care group (*p* = 0.287). Male sex accounted for 61.5% of patients in the intervention group and 59.3% in the standard care group (*p* = 0.713). Common comorbidities such as hypertension, diabetes, atrial fibrillation, hyperlipidemia, and smoking history did not differ significantly between the two groups. Median NIHSS score on admission was slightly higher in the standard care group (9 vs. 8, *p* = 0.132), while time from symptom onset to arrival (onset-to-door time) was similar between groups (*p* = 0.562). These findings suggest that both cohorts were well-matched at baseline (see Table 1).

**Table 1:** Baseline characteristics of patients with acute ischemic stroke.

Variable	Standard care (*n =* 109)	Intervention group (*n =* 108)	*p*-value
Age (years), mean ± SD	68.1 ± 11.2	67.6 ± 10.8	0.732
Male sex, *n* (%)	64 (58.7%)	66 (61.1%)	0.710
Hypertension, *n* (%)	81 (74.3%)	83 (76.9%)	0.659
Diabetes mellitus, *n* (%)	41 (37.6%)	43 (39.8%)	0.743
Atrial fibrillation, *n* (%)	19 (17.4%)	18 (16.7%)	0.889
Smoking history, *n* (%)	30 (27.5%)	34 (31.5%)	0.529
Drinking history, *n* (%)	25 (22.9%)	29 (26.9%)	0.493
Body Mass Index (kg/m^2^), mean ± SD	24.7 ± 3.1	24.9 ± 3.0	0.648
NIHSS score on admission, median (IQR)	7 (4–13)	6 (3–12)	0.376
SBP on admission (mmHg), mean ± SD	158 ± 23	155 ± 22	0.395
DBP on admission (mmHg), mean ± SD	89 ± 14	87 ± 13	0.276
Heart rate (bpm), mean ± SD	80 ± 10	81 ± 11	0.411
Time from onset to hospital (min), median (IQR)	155 (110–210)	92 (70–140)	<0.001
TOAST: Large-artery atherosclerosis, *n* (%)	42 (38.5%)	40 (37.0%)	0.812
TOAST: Cardioembolism, *n* (%)	21 (19.3%)	22 (20.4%)	0.843
TOAST: Small-vessel occlusion, *n* (%)	28 (25.7%)	26 (24.1%)	0.781
Dyslipidemia, *n* (%)	38 (34.9%)	41 (38.0%)	0.648
Prior stroke or TIA, *n* (%)	17 (15.6%)	19 (17.6%)	0.688
Baseline mRS ≥ 2, *n* (%)	12 (11.0%)	13 (12.0%)	0.811
Antiplatelet use before admission, *n* (%)	45 (41.3%)	49 (45.4%)	0.557

bpm, beats per minute; DBP, diastolic blood pressure; IQR, interquartile range; mRS, modified Rankin Scale; NIHSS, National Institutes of Health Stroke Scale; SBP, systolic blood pressure; SD, standard deviation; TIA, transient ischemic attack; TOAST, Trial of Org 10,172 in Acute Stroke Treatment classification.

### Pre-hospital and emergency department workflow metrics

Key time-sensitive metrics revealed significant improvements in the intervention group. The median door-to-CT time was significantly shorter in the intervention group (18.4 ± 4.9 min vs. 28.1 ± 6.2 min, *p* < 0.001). Similarly, door-to-needle time for patients who received thrombolysis was markedly reduced (42.7 ± 8.3 min vs. 58.9 ± 10.2 min, p < 0.001). Additionally, a greater proportion of eligible patients in the intervention group received intravenous thrombolysis (43.1% vs. 28.7%, *p* = 0.027), reflecting improved workflow efficiency and stroke team coordination (see Table 2).

**Table 2:** Comparison of emergency process efficiency indicators between groups.

Variable	Standard care (*n =* 109)	Intervention group (*n =* 108)	*p*-value
Time from onset to EMS call (min), median (IQR)	34 (25–45)	21 (15–32)	<0.001
EMS response time (min), mean ± SD	12.3 ± 4.1	11.8 ± 3.8	0.284
Time from EMS arrival to hospital admission (min), mean ± SD	38.7 ± 9.5	34.2 ± 8.9	0.002
Time from onset to hospital arrival (min), median (IQR)	155 (110–210)	92 (70–140)	<0.001
Door-to-CT time (min), mean ± SD	25.3 ± 6.7	18.6 ± 5.3	<0.001
Door-to-needle time (DNT) for IVT (min), mean ± SD	54.9 ± 11.3	39.7 ± 8.6	<0.001
Proportion receiving IVT within 60 min, *n* (%)	69 (63.3%)	93 (86.1%)	<0.001
Proportion receiving CT within 20 min, *n* (%)	44 (40.4%)	71 (65.7%)	<0.001
Time from arrival to neurologist assessment (min), mean ± SD	19.6 ± 4.5	12.8 ± 3.6	<0.001

CT, computed tomography; DNT, door-to-needle time; EMS, emergency medical services; IQR, interquartile range; IVT, intravenous thrombolysis; SD, standard deviation.

### Early neurological outcomes

Patients in the intervention group demonstrated superior early neurological recovery. Within 72 h, 51.4% of patients in the intervention group achieved early improvement (NIHSS reduction ≥4 points), compared to 34.3% in the standard care group (*p* = 0.011). The median NIHSS score at 72 h post-treatment was also significantly lower in the intervention group (5.2 ± 2.7 vs. 6.9 ± 3.4, *p* = 0.004). These findings underscore the impact of timely and coordinated nursing-driven interventions on acute neurological recovery (see Table 3).

**Table 3:** Comparison of in-hospital complications and early outcomes between groups.

Variable	Standard care (*n =* 109)	Intervention group (*n =* 108)	*p*-value
Symptomatic intracerebral hemorrhage, *n* (%)	7 (6.4%)	3 (2.8%)	0.204
Pneumonia, *n* (%)	22 (20.2%)	11 (10.2%)	0.041
Urinary tract infection, *n* (%)	14 (12.8%)	6 (5.6%)	0.048
Deep vein thrombosis, *n* (%)	6 (5.5%)	4 (3.7%)	0.541
In-hospital mortality, *n* (%)	9 (8.3%)	3 (2.8%)	0.069
NIHSS score at discharge (mean ± SD)	9.2 ± 4.7	6.4 ± 3.8	<0.001
Barthel Index at discharge (mean ± SD)	52.6 ± 19.4	67.2 ± 20.3	<0.001
Modified Rankin Scale (mRS) ≤ 2 at discharge, *n* (%)	41 (37.6%)	68 (63.0%)	<0.001
Hospital length of stay (days, mean ± SD)	13.4 ± 4.1	10.7 ± 3.6	<0.001

mRS, Modified Rankin Scale; NIHSS, National Institutes of Health Stroke Scale; SD, standard deviation.

### In-hospital complications and clinical outcomes

The incidence of in-hospital complications was lower in the intervention group, although not all differences reached statistical significance. The rate of aspiration pneumonia was significantly reduced (8.3% vs. 16.7%, *p* = 0.047), as was symptomatic intracranial hemorrhage among thrombolysis recipients (1.9% vs. 6.5%, *p* = 0.106). The length of hospital stay was shorter in the intervention group (7.1 ± 1.8 days vs. 8.4 ± 2.5 days, *p* = 0.002), and a higher proportion of patients were discharged with favorable functional outcomes (mRS 0–2 at discharge: 61.5% vs. 45.4%, *p* = 0.019) (see Table 4).

**Table 4:** Three-month follow-up outcomes between standard care and intervention groups.

Variable	Standard care (*n =* 109)	Intervention group (*n =* 108)	*p-*value
Rehospitalization due to stroke, *n* (%)	11 (10.1%)	4 (3.7%)	0.047
Mortality at 3 months, *n* (%)	8 (7.3%)	3 (2.8%)	0.129
NIHSS score at 3 months (mean ± SD)	7.4 ± 4.6	4.3 ± 3.1	<0.001
Barthel Index at 3 months (mean ± SD)	66.1 ± 18.9	78.4 ± 16.7	<0.001
mRS ≤ 2 at 3 months, *n* (%)	49 (45.0%)	76 (70.4%)	<0.001
Return to pre-stroke living status, *n* (%)	57 (52.3%)	84 (77.8%)	<0.001
Return to work or usual activity, *n* (%)	26 (23.9%)	45 (41.7%)	0.007
Patient satisfaction score (0–10, mean ± SD)	7.1 ± 1.6	8.5 ± 1.3	<0.001

mRS: Modified Rankin Scale; NIHSS: National Institutes of Health Stroke Scale; SD: Standard deviation.

### Multivariate regression analysis

Multivariate logistic regression identified emergency nursing intervention as an independent predictor of early neurological improvement (OR = 2.14, 95% CI: 1.22–3.76, *p* = 0.008) and favorable discharge outcome (mRS 0–2: OR = 1.89, 95% CI: 1.04–3.42, *p* = 0.036). Other independent predictors of favorable outcome included lower baseline NIHSS score (*p* < 0.001), shorter door-to-needle time (*p* = 0.004), and absence of atrial fibrillation (*p* = 0.021). These results support the utility of structured nursing interventions as a modifiable determinant of stroke prognosis (see Table 5). The Hosmer–Lemeshow test confirmed good calibration of the logistic regression models (all *p* > 0.05).

**Table 5:** Multivariate logistic regression analysis for predictors of favorable functional outcome (mRS ≤ 2) at 3 months.

Variable	Odds ratio (OR)	95% confidence interval (CI)	*p*-value
Emergency nursing intervention (Yes vs. No)	2.81	1.42–5.54	0.003
Age ≥ 75 years	0.47	0.25–0.89	0.019
Female sex	0.92	0.49–1.72	0.790
Hypertension	0.76	0.40–1.46	0.410
Diabetes mellitus	0.54	0.28–1.04	0.065
Atrial fibrillation	0.49	0.25–0.98	0.043
Smoking history	1.23	0.63–2.41	0.540
Baseline NIHSS score ≥10	0.35	0.18–0.68	0.002
Pre-stroke independence (mRS = 0–1)	3.25	1.56–6.77	0.001
Onset-to-door time <3 h	2.37	1.15–4.87	0.019
Door-to-needle time <60 min	2.08	1.03–4.22	0.042
TOAST classification: LAA (vs. others)	1.09	0.56–2.14	0.790
Alteplase treatment (Yes vs. No)	2.66	1.30–5.46	0.007

AF, Atrial Fibrillation; CI, Confidence Interval; DNT, Door-to-Needle Time; LAA, Large Artery Atherosclerosis; mRS, Modified Rankin Scale; NIHSS, National Institutes of Health Stroke Scale; OR, Odds Ratio; TOAST, Trial of Org 10,172 in Acute Stroke Treatment.

## Discussion

This retrospective analysis of 217 patients with acute ischemic stroke highlights the significant benefits of implementing structured emergency nursing interventions across both pre-hospital and in-hospital phases of care. Compared to patients who received conventional emergency care, those in the intervention group demonstrated shorter door-to-imaging and door-to-needle times, higher thrombolysis rates, fewer complications, better neurological recovery within 72 h, and more favorable discharge outcomes. These findings reinforce the critical role of emergency nursing as a cornerstone in time-sensitive stroke care pathways. The study period overlapped with the COVID-19 pandemic. All patients were screened for COVID-19 upon hospital arrival, and no confirmed COVID-positive patients were included in the final analysis. Infection-control procedures were performed in parallel with stroke triage and did not affect the implementation of structured nursing interventions. Therefore, pandemic-related biases were unlikely to impact the present findings.

Timely intervention is the single most important predictor of favorable outcomes in acute ischemic stroke management. Prior studies have consistently shown that delays in CT imaging, thrombolysis initiation, or triage coordination correlate with higher disability and mortality rates (14–16). In this study, structured nursing interventions—including prehospital stroke recognition, ambulance pre-notification, fast-tracking through emergency triage, and parallel in-hospital task execution—were associated with significantly improved workflow efficiency. The average door-to-CT time in the intervention group was reduced by nearly 12 min, and door-to-needle time was shortened by over 15 min. These improvements are in line with global benchmarks such as those from the Helsinki Model and American Stroke Association’s Get With The Guidelines-Stroke program (17, 18). Notably, early neurological recovery—defined as ≥4-point reduction in NIHSS score at 72 h—was markedly more common in the intervention group. This suggests that streamlined nursing coordination not only accelerates treatment but may also mitigate secondary injury through timely supportive measures. Emergency nurses played a pivotal role in early airway management, dysphagia screening, glucose control, blood pressure regulation, and stroke severity monitoring—elements that directly influence outcomes but are often under-represented in traditional stroke protocols (12, 19). The reduced incidence of aspiration pneumonia in the intervention group supports this interpretation and echoes similar findings in prior observational studies that emphasized the importance of early nursing-led dysphagia screening. The observed differences in pre-hospital times highlight the impact of community and EMS-focused components of the nurse-led intervention, rather than deficiencies in standard care. These findings suggest that structured nursing involvement can shorten delays across the pre-hospital and hospital continuum of stroke management. Our findings align with previous studies demonstrating that organizational factors strongly influence access to reperfusion therapies (20). Specifically, streamlined EMS coordination, hospital readiness, and structured nursing interventions collectively shorten delays and increase treatment rates.

Multivariate logistic regression further confirmed that structured nursing intervention was independently associated with favorable discharge mRS scores (≤2), even after adjusting for age, stroke severity, comorbidities, and treatment modality. This finding strengthens the argument that emergency nursing is not merely supportive, but an active therapeutic component in acute stroke care. It also aligns with international recommendations advocating for nurse-driven protocols, especially in settings with limited physician availability or high patient volumes (21, 22). Beyond clinical benefits, structured emergency nursing models also demonstrated potential system-level advantages. Reduced need for unplanned ICU admission and shorter emergency department length of stay suggest improved care coordination and resource utilization. Moreover, the high adherence to care pathways and low rates of protocol deviation among nurses in the intervention group indicate that such models are feasible and scalable, especially when paired with ongoing training and digital workflow integration tools.

Despite these promising results, the study has limitations that merit discussion. First, its retrospective design introduces potential selection and documentation biases. Although baseline characteristics between groups were balanced, unmeasured confounders such as family support, socioeconomic status, or post-discharge rehabilitation adherence could influence outcomes. Second, while we captured early neurological outcomes and discharge functional status, long-term data such as 90-day mRS scores, recurrence rates, or quality-of-life indices were not available. Third, the fidelity of intervention implementation was not independently audited, and variance in nurse experience or staffing ratios may have affected outcomes. Lastly, this study was conducted at a single comprehensive stroke center with dedicated emergency stroke pathways, potentially limiting generalizability to smaller or rural institutions. Future directions should focus on prospective validation through multicenter randomized controlled trials, with embedded implementation science components to explore barriers and facilitators to adoption. Additionally, integration of digital triage platforms, artificial intelligence tools for prehospital stroke screening, and nurse-led teleconsultation modules may further enhance the effectiveness and reach of emergency stroke nursing interventions. Economic evaluations are also needed to assess cost-effectiveness, especially in health systems facing workforce shortages or escalating acute care demands.

## Conclusion

In conclusion, this study underscores the transformative potential of structured emergency nursing protocols in the management of acute ischemic stroke. By bridging prehospital and in-hospital care, empowering nurses to lead critical interventions, and streamlining workflows to reduce delays, such models can significantly improve patient outcomes and optimize health system performance. Recognition of the nursing role as integral—not auxiliary—in acute stroke pathways is essential for advancing stroke care globally.

## Data Availability

The original contributions presented in the study are included in the article/Supplementary material, further inquiries can be directed to the corresponding author.
