# Supplementary material for: Impact of emergency nursing interventions on pre-hospital and in-hospital outcomes in acute ischemic stroke
Source: Front Neurol. 2025 Nov 24;16:1673515. doi: 10.3389/fneur.2025.1673515 (PMC12682623; doi:10.3389/fneur.2025.1673515)
Supplement: Supplementary file 1 [file Table_1.docx]

Supplementary Table 1: Comparison of Standard Care vs. Nurse-led Intervention.

| Aspect | Standard Care | Nurse-led Intervention |
| --- | --- | --- |
| Pre-hospital recognition | Public awareness, no structured program | Nurse-led community education and EMS training |
| EMS handover | Routine transport, no pre-notification | Pre-notification and streamlined handover by EMS nurses |
| ED triage | Physician-led, routine prioritization | Nurse-led triage with direct coordination for imaging |
| Documentation | Standard nursing records | Real-time monitoring of door-to-needle and door-to-CT times |
